# Accelerometer-measured physical activity and sample-based frailty in older women: does pattern really matter?

**DOI:** 10.3389/fpubh.2023.1304279

**Published:** 2024-01-25

**Authors:** Ting Li, Guanyang Zong, Pan Peng, Shiqiang Wang, Bin Cheng

**Affiliations:** ^1^School of Physical Education, Shandong University, Jinan, China; ^2^Dazhuang Primary School, Zoucheng, China; ^3^Ezhou High School, Ezhou, Hubei, China; ^4^College of Physical Education, Hunan University of Technology, Zhuzhou, China; ^5^Hunan Key Laboratory of Physical Health and Sports Fitness, Zhuzhou, China; ^6^Hunan Research Centre in Physical Fitness, Health, and Performance Excellence, Hunan University of Technology, Hunan, China

**Keywords:** community-dwelling older Chinese women, frailty, frailty subdomain, bouted PA, sporadic PA

## Abstract

**Background:**

The relationship between the patterns of physical activity (PA) and frailty, including its various subdomains, remains poorly understood. Therefore, this study aims to investigate the correlations between the patterns of physical activity and frailty and its various subdomains in community-dwelling older women.

**Methods:**

A cross-sectional study investigated the association between physical activity and frailty in 1,099 women aged between 60 to 70 years. Triaxial accelerometers were used to measure bouted PA (a minimum duration of 10 min) and sporadic PA (a duration of <10 min). Fried's frailty phenotype was utilized to evaluate the status of frailty. Data were analyzed using logistic regression and the receiver operating characteristic (ROC) curve.

**Results:**

Bouted moderate-to-vigorous PA (MVPA) and sporadic MVPA were associated with decreased odds of being prefrail and frail, and the optimal cutoff values were 6 and 19.7 for the prefrail stage and 6.6 and 19.4 min/day for the frail stage, respectively. Bouted light PA (LPA) was associated with decreased odds of being prefrail, and the optimal cutoff value was 170.2 min/day. Additionally, bouted and sporadic MVPA were associated with decreased odds of being slow and their optimal cutoff values were 5 and 19.1 min/day, respectively. Sporadic MVPA was associated with decreased odds of exhaustion, and the cutoff was 19.7 min/day. Bouted MVPA and LPA were associated with decreased odds of having low PA, and the cutoff values were 4.4 and 163.2 min/day, respectively.

**Conclusion:**

Any MVPA, regardless of bout duration, could be used as a suitable PA program to improve and prevent frailty in older women, such as bouted MVPA (4–5 times/week) or sporadic MVPA (20 min/day). The improvement effect of bouted and sporadic MVPA on the frailty of older people may not be affected by the subdomain. Additionally, bouted LPA was suitable for the management of prefrailty.

## Introduction

Frailty, characterized by reduced physiologic reserves, is linked with falls, disability, and mortality (1). With increased population aging, the number of people living with frailty is rapidly increasing worldwide (2), which would place increased pressure and challenge on the healthcare systems. In 2020, the number of individuals aged 60 years and above in China reached 260 million, representing 18.70% of its overall population. Among this group, ~7% of the community residents were affected by frailty (3). Multiple studies have demonstrated a higher prevalence of frailty in women, which is exacerbated by age (4). Studies revealed that the development of frailty is slow and dynamically reversible, and the effect of improving frailty in the early stage is more valid (5). Early and actively targeted interventions will more efficiently improve and reverse frailty.

Regular physical activity (PA) is crucial for enhancing the health of older adults living with frailty. Prior research has consistently shown a positive correlation between increased total PA time and frailty (6). Previous public health guidelines have traditionally recommended that older adults engage in 150 to 300 min of moderate-to-vigorous physical activity (MVPA) per week, with each activity lasting a minimum of 10 min at a time to reap health benefits. However, meeting such PA recommendations can be challenging for older adults living with frailty (7). A systematic review found that MVPA in bouts of ≥10 min in duration (bouted MVPA) and MVPA in bouts <10 min in duration (sporadic MVPA) were linked with beneficial health results, including BMI, body fat, and all-cause mortality (8), suggesting that participation in MVPA may have an enhancing effect regardless of the bout duration. Recently, the WHO guidelines had highlighted the potential health benefits of older adults engaging in intermittent or sporadic MVPA as compared to inactivity (9). Only a few studies, however, addressed the relationship between bouted and sporadic MVPA with physical frailty in older adults. Light PA (LPA) has been shown to be associated with mobility disability (10), heart disease (11), and other health outcomes in older women. However, it is not clear whether LPA is associated with frailty. Chen et al. (12) found that LPA in bouts of <10 min (sporadic LPA) was linked with a reduced risk of functional disability among older adults. Another study showed that both LPA in bouts ≥10 min (bouted LPA) and sporadic LPA in older men had a significant association with all-cause mortality (13). Further investigation is warranted for the potential feasibility of sporadic LPA as a means to ameliorate the frailty status in older individuals.

The frailty phenotype, as proposed by Fried, is defined as meeting at least three out of five subdomains: unintentional weight loss, slowness, weakness, low physical activity, and exhaustion. Those who meet 1–2 subdomains are considered prefrail (1). Frailty and prefrailty may represent varying degrees of decline in health status among older people, but each frailty subdomain reflected a different etiology and clinical feature (14). Thus, older individuals living with frailty or prefrailty may experience various subdomains (e.g., slowness and weakness vs. exhaustion and weight loss). Previous research suggested that managing frailty through physical activity requires considering various combinations of frailty subdomains (15). However, the relationship between objectively measured patterns of physical activity and frailty subdomains remains poorly understood. Therefore, this study aims to examine the correlations between bouted and sporadic PA and frailty and its corresponding subdomains, as well as analyze the optimal PA guideline to prevent frailty among older women residing in the community.

## Method

### Study population

Participants were recruited from the baseline survey of the Physical Activity and Health in Older Women Study (PAHIOWS) conducted between March 2021 and June 2021. The survey involved 1,370 older women aged between 60 and 70 years residing in the community. Other studies (15, 16) have already reported the particulars of PAHIOWS. In brief, women living in the community and aged between 60 and 70 years were included in the study if they were able to communicate freely, walk independently or with crutch assistance, and complete questionnaires, as well as provide informed consent. The exclusion criteria included failure to meet minimum accelerometer wearing standards (*N* = 148), missing data on demographic (*N* = 50), frailty subdomains (*N* = 72), and/or another questionnaire (*N* = 1). After excluding individuals with incomplete information, our final sample consisted of 1,099 community-dwelling older women. All participants consented in writing to take part in this study. This research was approved by the ethics committee of the School of Nursing and Rehabilitation at Shandong University, China (2021-R-067).

### Measures

#### Frailty phenotype

Given the smaller body size and age of the East Asian population in our study, we retained all five of the original components of the Fried frailty phenotype, as well as their methodology to produce population-specific cutoff points, which includes the following five subdomains: weight loss, exhaustion, weakness, slowness, and low physical activity. Scores of ≥3, 1–2, or 0 subdomains were considered frail, prefrail, and robust, respectively. Weight loss was defined as a self-report of unintentional weight loss of more than 3 kg in the previous year (17). Exhaustion was defined as participants answering “yes” to the question “Have you felt fatigued for no reason in the past month?” or “a moderate amount/most of the time” to the question “Did you have the feeling that everything you were doing was an effort?”. Weakness was defined as grip strength in the lowest 20% at baseline, adjusted for body mass index (BMI), and the cutoff point was ≤ 19.4 kg for the BMI of ≤ 23.2 kg/m^2^, ≤ 20.5 kg for the BMI of 23.3–25.2 kg/m^2^, ≤ 20.9 kg for the BMI of 25.3–27.4 kg/m^2^, and ≤ 20.7 kg for the BMI of >27.4 kg/m^2^. Slowness was defined as the time to walk 5 m in the slowest 20% at baseline, adjusted for standing height, and the cutoff point was ≥4.13 s for height ≤ 160 cm and ≥4.06 s for height >160 cm. Low physical activity was defined as the lowest 20% of physical activity energy expenditure as assessed by the International Physical Activity Questionnaire Short Form (IPAQ-S-C) (18).

#### Bouted and sporadic physical activity

The participants were instructed by trained personnel to wear the triaxial accelerometer (Actigraph wGT3X-BT, Pensacola, FL, USA) on their left hip for seven consecutive days, except when bathing, swimming, and sleeping. The Actigraph wGT3X-BT used in this study has been well examined for validity and accuracy (19, 20). Participants with a minimum of 4 valid days and the wear time of ≥10 h per day were included in the analyses (21). We used ActLife 6.13.4 to read and analyze 60-s epoch data. Non-wear time was defined as a minimum of 90 consecutive min of zero intensity counting without a maximum of 2 min of counting from 0 to 100. LPA was defined as 100–1,951 counts/minute (CPM), and MVPA was defined as ≥1,952 CPM. Bouted LPA and MVPA were defined as ≥10 consecutive min, allowing up to 2 min out of 10 to fall below the intensity threshold for LPA or MVPA. Sporadic LPA and MVPA as any accumulation of LPA or MVPA in <10 min. To derive an estimate of the time (minutes) spent in bouted LPA, sporadic LPA, bouted MVPA, and sporadic MVPA per day, values were averaged over the number of valid days.

#### Covariates

Demographic data related to community-dwelling older women (age, living alone, drinking, education, and comorbidity) were collected by questionnaires. Comorbidity was defined as having two or more diseases diagnosed through the self-report from a physician. Multi-frequency bioimpedance analysis using the Tanita MC-180 assessed weight, body mass index (BMI), and muscle mass (MM). The Mini Nutritional Assessment Short Form (MNA-SF) assessed nutritional status. The Athens Insomnia Scale (AIS) was used to assess insomnia. The health-related quality of life was evaluated with the Chinese version of the Visual Analog Scale in the European Quality of Life questionnaire (EQ-5D-VAS). Cognitive function was assessed by means of the Chinese adaptation of the Mini-Mental State Examination (MMSE).

### Statistical analysis

STATA SE version 16.0 (StataCorp, Texas, USA) and MedCalc version 20.0.13 (MedCalc Software, Ostend, Belgium) were used for analyzing data. We described the mean sociodemographic and PA-related characteristics of the sample and expressed them as the main and standard deviation. Categorical variables were expressed in the form of frequencies and percentages. The Kruskal–Wallis and Jonckheere-Terpstra trend tests were used to analyze differences across groups. We analyzed associations between frailty status and its subdomains and different patterns of PA using the logistic regression analysis. In addition, to overcome the issue that low PA may increase the probability to find association, we preformed sensitivity analyses (including the main analyses as well as the subdomain and ROC analyses) while removing people with “low PA” and only considering four domains (weight loss, exhaustion, weakness, and slowness) to categorize subjects as either frail or prefrail. The results are expressed in the form of odds ratios (ORs) with their 95% confidence intervals (CIs). The following two models were used to adjust for confounding factors. Model 1 was adjusted for age, BMI, education, living alone, comorbidity, MM, drinking status, MMSE score, AIS score, MNA-SF score, EQ-5D-VAS score, and accelerometer wear time. Model 2 was additionally adjusted for total sedentary behavior (SB) time to bouted or sporadic MVPA (model 2a) and total MVPA time to bouted or sporadic LPA (model 2b). Model 3 included factors mentioned in model 2 as well as bouted MVPA to sporadic MVPA and vice versa (model 3a) and bouted LPA to sporadic LPA and vice versa (model 3b). Collinearity was detected by calculating the variance inflation factor (VIF) for all variables. In the fully adjusted model, the VIF for each covariate is below 5, which is acceptable. LPA was adjusted using total MVPA time (12). When the logistic regression analysis demonstrated significant correlations, the ROC analysis was performed to determine the optimal cutoff value for the patterns of PA variables in order to differentiate between frailty and frailty subdomains using the area under the curve (AUC) (22). Statistical significance was set at a *P-*value of <0.05.

## Results

In total, 1,099 community-dwelling older women were involved in the study. The mean age was 64.9 ± 2.8 years old. The characteristics, prevalence of frailty and prefrailty, and distribution of frailty subdomains for all participants are described in Table 1. According to the FP measurement, 5.6% of the participants had frailty and 52.6% had prefrailty. Compared with robust older women, women living with frailty were older, had insomnia, had malnutrition, had higher BMI, less muscle mass, poorer self-perceived health status, and had more comorbidity. Meanwhile, bouted and sporadic MVPA time were higher in robust older women.

**Table 1:** Characteristics of participants according to the frailty status.

**Variable**	**Total (*n* = 1,099)**	**Frailty status**			***P*-value**
		**Robust (*****n*** = **459)**	**Prefrailty (*****n*** = **578)**	**Frailty (*****n*** = **62)**	
Age, year	64.9 ± 2.8	64.5 ± 2.8	65.2 ± 2.8	65.8 ± 2.8	**<0.001**
BMI	25.4 ± 3.3	25.0 ± 3.0	25.6 ± 3.4	25.7 ± 3.9	**0.021**
Living alone	121 (11.1)	53 (11.5)	59 (10.2)	9 (14.5)	0.524
**Education**					
Primary or below	130 (11.8)	46 (10.0)	74 (12.8)	10 (16.1)	0.651
Junior or senior high	768 (69.8)	327 (71.2)	401 (69.4)	40 (64.5)	
College or above	201 (18.3)	86 (18.7)	103 (17.8)	12 (19.4)	
Current drinker	103 (9.4)	44 (9.5)	54 (9.3)	5 (8.1)	0.928
Comorbidity, ≥2	215 (19.6)	71 (15.5)	127 (22.0)	11 (27.4)	**0.002**
MM, kg	38.7 ± 2.8	38.9 ± 2.7	38.7 ± 2.9	37.5 ± 2.4	**0.001**
Handgrip	24.1 ± 5.0	25.6 ± 3.4	23.3 ± 5.7	19.6 ± 4.1	**<0.001**
Gait speed	3.8 ± 0.6	3.5 ± 0.4	3.9 ± 0.7	4.6 ± 0.9	**<0.001**
PA (IPAQ)	3,587.9 ± 1,925.6	4,209.2 ± 1,598.3	3,245.0 ± 2,017.6	2,186.0 ± 1,744.2	**<0.001**
MMSE score	26.7 ± 1.4	26.6 ± 1.4	26.7 ± 1.4	26.7 ± 1.3	0.495
AIS score	1.0 ± 2.2	0.6 ± 1.5	1.0 ± 2.3	3.1 ± 4.0	**<0.001**
MNA score	13.3 ± 1.1	13.4 ± 0.9	13.3 ± 1.1	12.9 ± 1.5	**0.036**
EQ-5D-VAS score	78.2 ± 12.9	80.8 ± 11.7	77.2 ± 12.6	68.1 ± 17.4	**<0.001**
**Physical activity**					
Wear time, min/day	887.6 ± 117.6	886.9 ± 110.3	884.6 ± 119.8	920.0 ± 143.0	0.117
Bouted MVPA time, min/day	11.8 ± 13.2	14.4 ± 14.1	10.5 ± 12.3	5.9 ± 9.9	**<0.001**
Sporadic MVPA time, min/day	20.9 ± 11.0	23.7 ± 10.8	19.5 ± 10.8	14.6 ± 8.8	**<0.001**
Bouted LPA time, min/day	181.7 ± 74.4	188.7 ± 77.6	176.8 ± 71.4	176.2 ± 73.8	0.275
Sporadic LPA time, min/day	125.5 ± 23.1	126.8 ± 21.9.6	124.7 ± 23.9	123.3 ± 24.5	0.054
**Frailty subdomains**					
Weight loss	113 (10.3)	0 (0)	91 (15.7)	22 (35.5)	–
Exhaustion	191 (17.3)	0 (0)	144 (24.9)	47 (75.8)	–
Weakness	223 (20.3)	0 (0)	181 (31.3)	44 (71.0)	–
Slowness	225 (20.4)	0 (0)	173 (29.9)	50 (80.6)	–
Low physical activity	220 (20.1)	0 (0)	184 (31.8)	36 (58.1)	–

Data show mean ± SD or n (%). BMI, body mass index; MM, muscle mass; MMSE, Mini-Mental State Examination; AIS, Athens Insomnia Scale; MNA-SF, Mini Nutritional Assessment Short Form; EQ-5D-VAS, EuroQol-5D Visual Analog Scale; LPA, light physical activity; MVPA, moderate-to-vigorous physical activity. Bold values denote *P* < 0.05.

**Figure 1:**
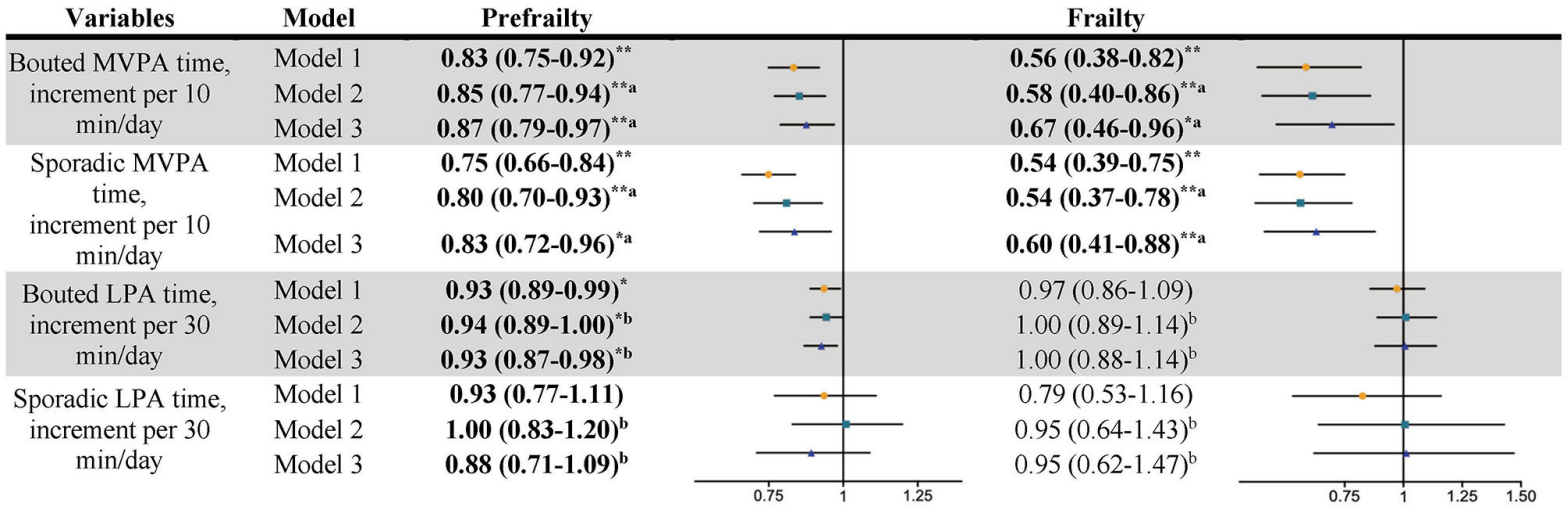
Association between the pattern of PA and frailty status. Data shows odds ratio and 95% confidence interval. Model 1, adjusted for wear time, age, BMI, education, living alone, drinking status, Comorbidity, MM, MMSE score, AIS score, MNA score, and EQ-5D-VAS score. Model 2, a, additional adjusted total SB time; b additional adjusted total MVPA time; Model 3, a, additional adjusted bouted MVPA and sporadic MVPA; b, additional adjusted bouted LPA and sporadic LPA. *P < 0.05; **P < 0.01. *n* = 1,099. Bold values denote *P* < 0.05.

**Figure 2:**
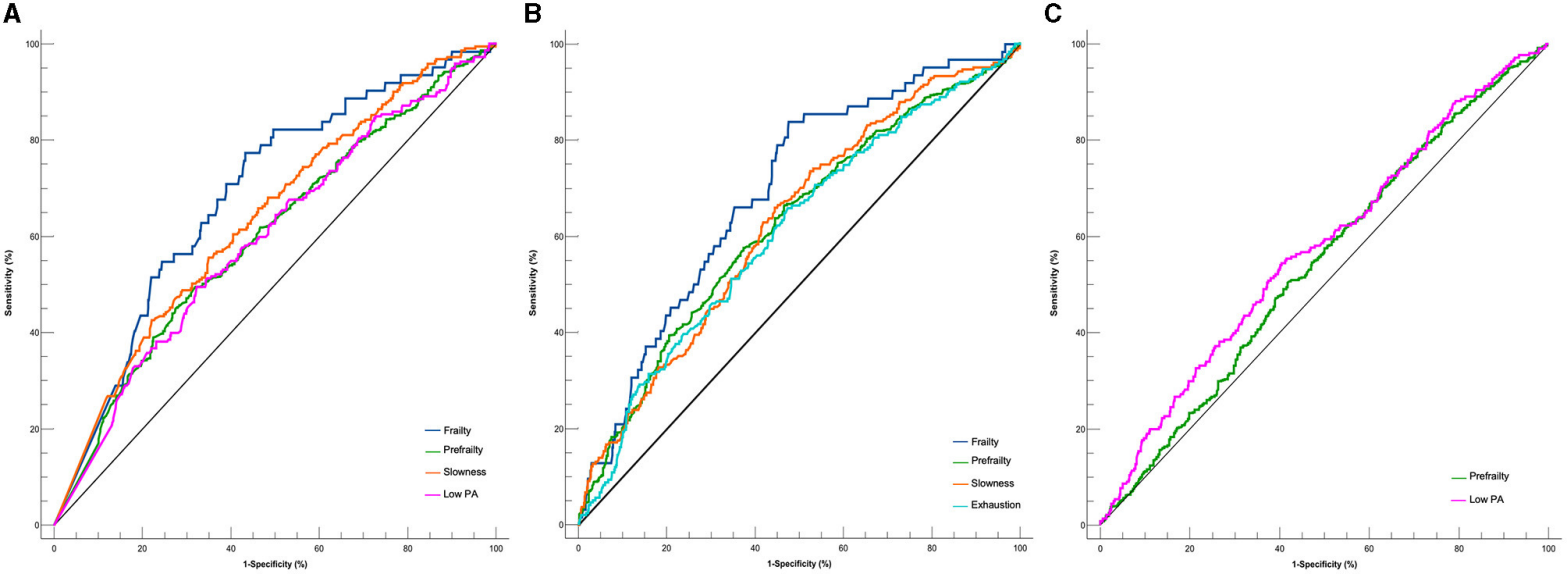
Optimal cutoffs of pattern of PA for screening frailty status and subdomains. **(A)** Bouted MVPA. **(B)** Sporadic MVPA. **(C)** Sporadic LPA.

Regarding associations between the patterns of PA and frailty status (Figure 1), each additional 10-min increase in bouted and sporadic MVPA was associated with 13% (0.87, 95% CI: 0.79–0.97) and 17% (0.83, 95% CI: 0.72–0.96) decreases in the ORs of being prefrail, respectively. Every 30-min increase in bouted LPA was associated with a 7% (0.93, 95% CI: 0.87–0.98) decrease in the ORs of being pre-frail in model 3. The optimal cutoff values to discriminate prefrailty were 6-min bouted MVPA per day (amounting to ~4.2 times per week), 19.7-min sporadic MVPA per day, and 170.2-min bouted LPA per day (Table 2 and Figure 2). Only every 10-min increase in bouted and sporadic MVPA was associated with 33% (0.67, 95% CI: 0.46–0.96) and 40% (0.60, 95% CI: 0.41–0.88) decreases in the ORs of being frail, respectively. The optimal cutoff values to discriminate frailty were 6.6-min bouted MVPA per day (amount to about 4.6 times per week) and 19.4-min sporadic MVPA per day (Table 2). Sporadic LPA was not associated with any frailty status. Consistent with the results of the above analyses, sensitivity analyses also showed that MVPA patterns were associated with decreased the ORs of being prefrail or frail, except for the fact that the association between LPA and frailty status disappeared (Supplementary Tables S1, S3).

**Table 2:** Optimal cutoff values of patterns of PA for screening frailty status and subdomains.

	**Cutoff (min/day)**	**AUC (95% CI)**	**Sensitivity (%)**	**Specificity (%)**	***P*-value**
**Bouted MVPA time**					
Prefrailty	≤ 6	0.60 (0.57–0.63)	49.13	68.41	<0.001
Frailty	≤ 6.6	0.69 (0.66–0.71)	77.42	56.61	<0.001
Slowness	≤ 5	0.64 (0.61–0.67)	55.61	65.07	<0.001
Low PA	≤ 4.4	0.60 (0.57–0.63)	49.55	67.69	<0.001
**Sporadic MVPA time**					
Prefrailty	≤ 19.7	0.62 (0.59–0.65)	57.44	62.75	<0.001
Frailty	≤ 19.4	0.69 (0.66–0.72)	83.87	52.36	<0.001
Exhaustion	≤ 19.7	0.61 (0.58–0.64)	65.97	52.75	<0.001
Slowness	≤ 19.1	0.63 (0.60–0.66)	66.37	55.48	<0.001
**Bouted LPA time**					
Prefrailty	≤ 170.2	0.54 (0.51–0.57)	50.52	58.17	0.017
Low PA	≤ 163.2	0.58 (0.55–0.61)	54.55	59.50	<0.001

AUC, area under the curve.

**Figure 3:**
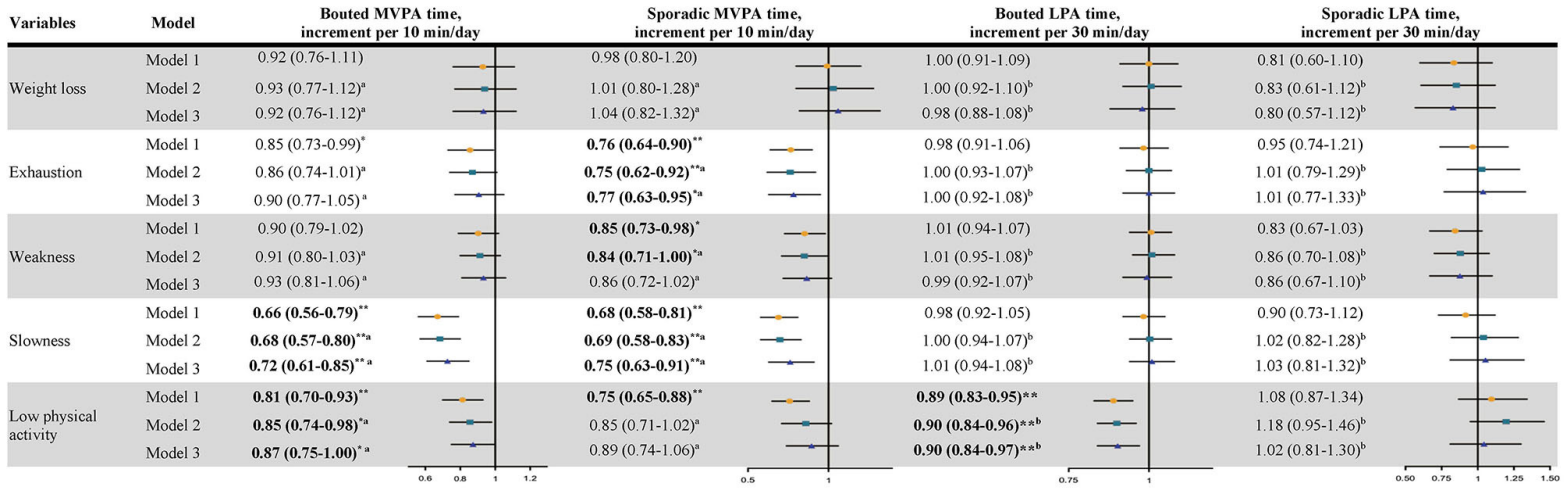
Association between the pattern of PA and frailty subdomains. Data shows odds ratio and 95% confidence interval. Model 1, adjusted for wear time, age, BMI, education, living alone, drinking status, Comorbidity, MM, MMSE score, AIS score, MNA score, and EQ-5D-VAS score. Model 2, a, additional adjusted total SB time; b additional adjusted total MVPA time; Model 3, a, additional adjusted bouted MVPA and sporadic MVPA; b, additional adjusted bouted LPA and sporadic LPA. *P < 0.05; **P < 0.01. *n* = 1,099. Bold values denote *P* < 0.05.

Figure 3 shows the results of patterns of PA on single frailty subdomain. A multivariate analysis showed that only an increase of 10 min in sporadic MVPA was associated with a 23% (0.77, 95% CI: 0.63–0.95) decrease in the ORs of exhaustion after adjusting for all confounders, and the optimal cutoff value was 19.7 min/day (Table 2 and Figure 2). After an additional adjustment for the bouted MVPA, sporadic MVPA was not related to weakness. Each additional 10-min increase in bouted and sporadic MVPA was associated with 28% (0.72, 95% CI: 61–0.85) and 25% (0.75, 95% CI: 0.63–0.91) decreases in the ORs of slowness, respectively. The optimal cutoff values to discriminate slowness were 5-min bouted MVPA per day (amount to about 3.5 times per week) and 19.1-min sporadic MVPA per day. Every 10-min increase in bouted MVPA and 30-min increase in bouted LPA were associated with 13% (0.87, 95% CI: 0.75–1.00) and 10% (0.90, 95% CI: 0.84–0.97) decreases in the ORs of having low PA, respectively. The optimal cutoff values to discriminate low PA were 4.4-min bouted MVPA per day (amount to about three times per week) and 163.2-min bouted LPA per day. Weight loss was not associated with any patterns of PA. Consistent with the results of the above analyses, sensitivity analyses also showed that the MVPA patterns were associated with decreased ORs of exhaustion or slowness, except the LPA patterns (Supplementary Tables S2, S3).

## Discussion

To the best of our knowledge, this is the first study to reveal an association between objectively measured bouted and sporadic PA and frailty status and its subdomains. We found that bouted MVPA was associated with decreased odds of being prefrail, frail, slowness, and low PA, while sporadic MVPA was associated with decreased odds of being prefrail, frail, exhaustion, and slowness. Bouted LPA was only associated with decreased odds of being prefrail and low PA subdomain. In addition, our results suggest that bouted MVPA of the week such as 30–46 min (4.4–6.6 min over 7 days) in bouts of a minimum of 10 min or sporadic MVPA of 20 min/day may be more beneficial for the management of frailty.

Our research revealed that both sporadic and bouted MVPA exhibit a connection with prefrailty and frailty that is independent of confounding variables after full adjustment. The ROC analysis also showed consistent results, with an optimal cutoff value for distinguishing between prefrailty and frailty being ~5–7-min bouted MVPA per day (amounting to ~3–4.6 times per week) (*P* < 0.001), which is slightly lower than that in the study of Chen et al. (23) (9.13 min/day). The optimal cutoff values for sporadic MVPA to distinguish between prefrailty and frailty were ~20 min/day (*P* < 0.001), almost consistent with the WHO recommendations for PA in older adults. The information we provided corroborates the current guidelines, indicating that the MVPA of any duration can lead to better health outcomes, including a reduction in frailty (8, 24). Additionally, there are established associations between sporadic MVPA and lower bouted MVPA time (only 7 min per day) with a frailty status. Older people can get health benefits from MVPA bouts, regardless of the duration. However, based on the ROC results, we speculated that the impact prolonged activity may have on someone with low physical function, may discourage engagement and that bouted MVPA would be more suitable for them. Meanwhile, our study found that prefrailty was associated with bouted LPA, of which bouts of 170.2 min/day could significantly distinguish prefrailty. However, some studies have concluded that LPA was not associated with the risk of frailty (23, 25), and our sensitivity analysis also found that the association between LPA patterns and frailty status disappeared after excluding participants with low PA, which could be due to the fact that the frailty subdomain, i.e., low PA, may increase the association between frailty and LPA patterns, and the association between LPA and prefrailty/frailty was explored due to the decrease in the sample size. In our previous results, we found that bouted LPA was only associated with prefrailty. However, to understand the diverse effects of sporadic and bouted LPA on frailty status, there is a need for more high-quality and prospective studies in the future.

Meanwhile, we further investigated the associations between bouted and sporadic PA and the frailty subdomains. Both main analysis and sensitivity analysis found that sporadic MVPA, instead of bouted MVPA, was significantly associated with exhaustion. In general, older adults are more likely to frequently participate in short-duration and sporadic MVPA than tiring and bouted MVPA. Our study also showed from this result that the average time of bouted MVPA in older women was ~12 min/day and the time of sporadic MVPA was ~21 min/day, which was nearly twice as long as bouted MVPA. The increased total energy expenditure associated with prolonged sporadic MVPA (26) may be one reason for improved exhaustion. After fully adjusting confounding factors, we found that any MVPA, regardless of the bout duration, was negatively associated with a lower risk of slowness, as were the results of the sensitivity analysis. A previous study found that long-term moderate to high-intensity aerobic exercise could reduce age-related muscle strength decline (27) and effectively improve the slowness of older women. It is tempting to speculate that bouted PA was associated with low PA rather than sporadic PA, which is related to the evaluation method of low PA. The study found that the IPAQ, which consists of bouted MVPA, overestimated the amount of bouted MVPA, and this was likely due to older women tending to recall long-term PA easily and reported MVPA bouts that were <10 continuous min (28).

Our results showed that the optimal cutoff values of bouted MVPA to differentiate between slowness and low PA were 5 and 4.4 min/day, respectively, which were not significantly different from the value to distinguish frailty. The optimal cutoff values of sporadic MVPA to differentiate between exhaustion and slowness were 19.7 and 19.1 min/day, respectively, which were similar to those for distinguishing frailty. The optimal cutoff values of bouted LPA for distinguishing low PA and prefrailty were basically the same (163.2 min/day), which is different from the result from a previous investigation on the total MVPA time corresponding to different frailty subdomains (15), which found that the optimal cutoff values of total MVPA time corresponding to distinguish frailty subdomains were different, ranging from 20 to 30 min/day. However, in this study, the cutoff values to distinguish each subdomain or frailty status were almost the same, 5–7-min bouted MVPA per day (amounting to ~3–4.6 times per week) and 20-min sporadic MVPA per day. The results may indicate that the improvement effect of bouted and sporadic MVPA on the frailty of older people may not be affected by the subdomain when considering the patterns of MVPA alone.

### Limitation

First of all, because the population of this study is from a city in China involving women aged between 60 and 70 years, it cannot represent the results of the entire Chinese population. Second, we used a cross-sectional study to collect participant information and could not infer cause and effect in the study, which means that older women could not be active because of frailty. This causal relationship needs to be proven in further studies, and it should be noted that we used sample-based cutoffs rather than raw cutoffs to assess frailty. Although most confounders were adjusted for in this study, there may be other residual confounders. In addition, participants who volunteered to participate in the study at community centers were likely to be more health-conscious and in better physical shape than those who did not participate.

## Conclusion

In summary, bouted and sporadic MVPA were associated with decreased odds of being frail and may serve as PA recommendations for improving frailty in community-dwelling older Chinese women. Bouted LPA was only suitable for the management of prefrailty. Considering the patterns of MVPA alone, the effect of MVPA on frailty may not be affected by subdomains. The optimal cutoff values of bouted and sporadic MVPA in differentiating frailty and its subdomains were almost the same in older women. Bouted MVPA of 5–7 min/day or sporadic MVPA of 20 min/day may be more conducive to frailty management, while an LPA of 170.2 min/day may be only valid for prefrailty.

## Data availability statement

The datasets presented in this article are not readily available because research data are not shared. Requests to access the datasets should be directed at: liting19980208@163.com.

## Ethics statement

This research was approved by the Ethics Committee of the School of Nursing and Rehabilitation, Shandong University, China (2021-R-067). The studies were conducted in accordance with the local legislation and institutional requirements. The participants provided their written informed consent to participate in this study.

## Author contributions

TL: Investigation, Methodology, Writing—original draft. GZ: Data curation, Investigation, Writing—original draft. PP: Data curation, Supervision, Writing—review & editing. SW: Writing—review & editing. BC: Writing—review & editing.
