# Supplementary material for: Accelerometer-measured physical activity and sample-based frailty in older women: does pattern really matter?
Source: Front Public Health. 2024 Jan 25;11:1304279. doi: 10.3389/fpubh.2023.1304279 (PMC10850322; doi:10.3389/fpubh.2023.1304279)
Supplement: Supplementary file 1 [file Table_1.DOCX]

Supplementary Material

# Supplementary Tables

| **Table S1 Associations between the pattern of PA and Frailty Status in samples that exclude participants with low PA** | | | |
| --- | --- | --- | --- |
| **Variables** | **Model 1** | **Model 2** | **Model 3** |
| Bouted MVPA time,  increment per 10 min/day | **0.83 (0.74-0.93)^**^** | **0.85 (0.75-0.95)^**a^** | **0.88 (0.78-0.98)^*a^** |
| Sporadic MVPA time,  increment per 10 min/day | **0.73 (0.63-0.84)^**^** | **0.75 (0.65-0.88)^**a^** | **0.78 (0.67-0.92)^*a^** |
| Bouted LPA time,  increment per 30 min/day | 0.97 (0.91-1.02) | 0.97 (0.92-1.03)^b^ | 0.95 (0.89-1.02)^b^ |
| Sporadic LPA time,  increment per 30 min/day | 0.86 (0.71-1.06) | 0.94 (0.77-1.15)^b^ | 0.87 (0.69-1.10)^b^ |
| Note: Data shows Odds ratio and 95% confidence interval. Model 1, adjusted for wear time, age, BMI, education, living alone, drinking status, Comorbidity, MM, MMSE score, AIS score, MNA score, and EQ-5D-VAS score. Model 2, a, additional adjusted total SB time; b, additional adjusted total MVPA time; Model 3, a, additional adjusted bouted MVPA and sporadic MVPA; b, additional adjusted bouted LPA and sporadic LPA. *, P<0.05; **, P<0.01. n=1099. | | | |

| **Table S2 Associations between the pattern of PA and Frailty Subdomains in samples that exclude participants with low PA** | | | | |
| --- | --- | --- | --- | --- |
| **Variables** | **Model 1** | **Model 2** | | **Model 3** |
| Bouted MVPA time, increment per 10 min/day |  |  |  | |
| Weight loss | 0.94 (0.77-1.14) | 0.93 (0.76-1.14)^a^ | 0.94 (0.76-1.15)^a^ | |
| Exhaustion | 0.87 (0.73-1.02) | 0.88 (0.74-1.04)^a^ | 0.91 (0.77-1.08) ^a^ | |
| Weakness | 0.92 (0.80-1.06) | 0.94 (0.82-1.09)^a^ | 0.97 (0.84-1.11) ^a^ | |
| Slowness | **0.68 (0.57-0.82)^**^** | **0.70 (0.58-0.84)^**a^** | **0.74 (0.62-0.87)^** a^** | |
| **Sporadic MVPA time, increment per 10 min/day** |  |  |  | |
| Weight loss | 0.97 (0.77-1.22) | 0.96 (0.75-1.25)^a^ | 0.98 (0.75-1.28)^a^ | |
| Exhaustion | **0.77 (0.63-0.94)^*^** | **0.77 (0.61-0.97)^*a^** | **0.79 (0.63-0.99)^*a^** | |
| Weakness | 0.79 (0.66-0.94)^**^ | 0.82 (0.67-1.00)^*a^ | 0.83 (0.68-1.01)^a^ | |
| Slowness | **0.68 (0.58-0.82)^**^** | **0.70 (0.57-0.85)^**a^** | **0.75 (0.61-0.93)^**a^** | |
| **Bouted LPA time, increment per 30 min/day** |  |  |  | |
| Weight loss | 1.03 (0.93-1.04) | 1.03 (0.93-1.14)^b^ | 1.00 (0.90-1.12)^b^ | |
| Exhaustion | 0.98 (0.91-1.06) | 0.99 (0.91-1.08)^b^ | 0.99 (0.91-1.09)^b^ | |
| Weakness | 0.97 (0.89-1.04) | 0.97 (0.90-1.05)^b^ | 0.94 (0.87-1.03)^b^ | |
| Slowness | 0.98 (0.91-1.05) | 0.99 (0.92-1.06)^b^ | 0.99 (0.91-1.08)^b^ | |
| **Sporadic LPA time, increment per 30 min/day** |  |  |  | |
| Weight loss | 0.79 (0.56-1.10) | 0.80 (0.56-1.12)^b^ | 0.80 (0.54-1.17)^b^ | |
| Exhaustion | 0.95 (0.73-1.25) | 1.01 (0.77-1.34)^b^ | 1.01 (0.74-1.37)^b^ | |
| Weakness | 0.86 (0.67-1.10) | 0.90 (0.70-1.16)^b^ | 0.83 (0.62-1.10)^b^ | |
| Slowness | 0.92 (0.72-1.17) | 1.04 (0.81-1.35)^b^ | 1.03 (0.78-1.38)^b^ | |
| Note: Data shows Odds ratio and 95% confidence interval. Model 1, adjusted for wear time, age, BMI, education, living alone, drinking status, Comorbidity, MM, MMSE score, AIS score, MNA score, and EQ-5D-VAS score. Model 2, a, additional adjusted total SB time; b, additional adjusted total MVPA time; Model 3, a, additional adjusted bouted MVPAand sporadic MVPA; b, additional adjusted bouted LPA and sporadic LPA. *, P<0.05; **, P<0.01. n=1099. | | | | |

| **Table S3 Optimal Cutoffs of pattern of PA for Screening Frailty Status and Subdomains** | | | | | |
| --- | --- | --- | --- | --- | --- |
|  | Cutoff  (min/day) | AUC (95% CI) | Sensitivity (%) | Specificity (%) | *P* |
| **Bouted MVPA time** |  |  |  |  |  |
| Prefrailty/frailty | ≤ 6.2 | 0.60 (0.57-0.63) | 49.76 | 67.97 | **<.001** |
| Slowness | ≤ 5 | 0.63 (0.60-0.66) | 52.57 | 68.18 | **<.001** |
| **Sporadic MVPA time** |  |  |  |  |  |
| Prefrailty/frailty | ≤ 19.7 | 0.62 (0.59-0.65) | 58.10 | 62.75 | **<.001** |
| Exhaustion | ≤ 19.7 | 0.60 (0.57-0.63) | 62.68 | 55.77 | **<.001** |
| Slowness | ≤ 21.4 | 0.63 (0.60-0.66) | 70.86 | 50.00 | **<.001** |
| AUC, Area under the curve. | | | | | |
